# Influencing Factors on Pupillary Light Responses as a Biomarker for Local Retinal Function in a Large Normative Cohort

**DOI:** 10.1167/iovs.65.6.3

**Published:** 2024-06-03

**Authors:** Ricarda Jendritza, Krunoslav Stingl, Torsten Strasser, Ronja Jung, Felix Tonagel, Paul Richter, Anton Sonntag, Tobias Peters, Helmut Wilhelm, Barbara Wilhelm, Carina Kelbsch

**Affiliations:** 1 Pupil Research Group at the Centre for Ophthalmology, University of Tuebingen, Tuebingen, Germany; 2 University Eye Hospital, Centre for Ophthalmology, University of Tuebingen, Tuebingen, Germany; 3 Center for Rare Eye Diseases, University of Tuebingen, Tuebingen, Germany; 4 Institute for Ophthalmic Research, Centre for Ophthalmology, University of Tuebingen, Tuebingen, Germany

**Keywords:** pupillary light response (PLR), cone function, rod function, chromatic pupil campimetry (CPC)

## Abstract

**Purpose:**

Investigating influencing factors on the pupillary light response (PLR) as a biomarker for local retinal function by providing epidemiological data of a large normative collective and to establish a normative database for the evaluation of chromatic pupil campimetry (CPC).

**Methods:**

Demographic and ophthalmologic characteristics were captured and PLR parameters of 150 healthy participants (94 women) aged 18 to 79 years (median = 46 years) were measured with L-cone- and rod-favoring CPC protocols. Linear-mixed effects models were performed to determine factors influencing the PLR and optical coherence tomography (OCT) data were correlated with the pupillary function volume.

**Results:**

Relative maximal constriction amplitude (relMCA) and latency under L-cone- and rod-favoring stimulation were statistically significantly affected by the stimulus eccentricity (*P* < 0.0001, respectively). Iris color and gender did not affect relMCA or latency significantly; visual hemifield, season, and daytime showed only minor influence under few stimulus conditions. Age had a statistically significant effect on latency under rod-specific stimulation with a latency prolongation ≥60 years. Under photopic and scotopic conditions, baseline pupil diameter declined significantly with increasing age (*P* < 0.0001, respectively). Pupillary function volume and OCT data were not correlated relevantly.

**Conclusions:**

Stimulus eccentricity had the most relevant impact on relMCA and latency of the PLR during L-cone- and rod-favoring stimulation. Latency is prolonged ≥60 years under scotopic conditions. Considering the large study collective, a representative normative database for relMCA and latency as valid readout parameters for L-cone- and rod-favoring stimulation could be established. This further validates the usability of the PLR in CPC as a biomarker for local retinal function.

Chromatic pupil campimetry (CPC) allows for an objective, non-contact functional assessment of the photoreceptor system by measuring the pupillary light response (PLR) to focal light stimuli. In contrast to classic perimetry, CPC is independent of the patient's subjective perception and performance. Furthermore, it includes gaze-tracking to ensure retinotopic stimulation even in patients with severe fixation difficulties.^1^ Specific stimulation protocols have been implemented to separately stimulate cones and rods and to measure certain parameters of the PLR as biomarkers to characterize local retinal function.^2^^–^^5^

Photoreceptor dysfunction in patients with retinitis pigmentosa (RP) can be objectified by evaluating the reduced PLR in multifocal pupil campimetry (white stimuli on a flat screen)^6^ and chromatic pupillometry (red/blue stimuli).^7^^,^^8^ Previous studies have shown the value of PLR to full-field chromatic stimulation to quantify the residual photoreceptor function in patients with *CNGA3*-linked achromatopsia^9^ or advanced RP with non-recordable electroretinogram.^10^^–^^12^ CPC is even feasible to quantify the effect of Voretigene neparvovec on local rod and cone function in patients with bi-allelic *RPE65* mutation induced retinal dystrophies.^13^ CPC can be complemented by cell-specific transcorneal electrostimulation to investigate the residual neuronal function in retinal degenerations.^14^^–^^16^

Due to retinotopic and photoreceptor specific stimulation in CPC, the loss of central vision in Stargardt disease can be correlated with reduced cone function in the macular region with preserved yet altered rod function in terms of signal processing.^17^ In exudative age-related macular degeneration (AMD), previous findings have shown altered PLR^18^ and improvements in response dynamics in multifocal pupillography after the application of Ranibizumab.^19^ CPC reveals impaired cone function in the fovea with differentiation in disease activity of AMD by latency dynamics.^20^

Pupil campimetry has been used to objectify visual field (VF) defects in pathologies along the visual pathway^21^ and to further investigate the retrogeniculate pupillary pathway.^22^ Through specific stimulus characteristics, chromatic pupillography confirms the existence of an occipital in addition to a subcortical pupillomotor pathway^23^ and the preservation of the latter despite dorsal midbrain lesions.^24^ CPC can further particularize the impact of pre- and postchiasmal lesions on the PLR as compared to non-affected VF sections.^25^

Hence, CPC and comparable chromatic pupillometries are of particular interest in numerous ophthalmologic pathologies, especially retinal degenerations. They represent a complementary objective tool to morphologic retinal imaging as well as electrophysiological and psychophysical testing to monitor local retinal function in the natural course of diseases and during therapy. Knowledge about factors influencing the PLR is crucial for the evaluation of pupillary responses.

The aim of this study was to collect epidemiological data from a large collective of healthy participants to determine characteristics influencing the PLR and to establish a normative database for CPC which is of fundamental importance for the comparison with patients’ results in daily clinical use.

## Methods

### Study Design

The study obeyed the tenets of the Declaration of Helsinki and was approved by the ethics committee of the Faculty of Medicine, University of Tuebingen (775/2016B02 Amendment 2). All participants received detailed information about the study and gave their written consent.

One hundred fifty (150) volunteers (94 women) aged 18 to 79 years (median = 46 years) were divided into 5 equally sized age groups: 18 to 29, 30 to 39, 40 to 49, 50 to 59, and 60 to 79 years. The time of measurement was categorized in the “morning” (8–12 AM), the “afternoon” (12-6 PM), the “summer” (April-September), and “winter” (October-March).

Exclusion criteria were a refractive error larger than ± 3 diopters in sphere, cylinder, or spherical equivalent, best corrected visual acuity (BCVA) of less than 0.8, protanomaly/protanopia, optic media opacity, for example, corneal haze or advanced cataract, pupillary disorders, retinal diseases, or diseases of the optic nerve, such as, glaucoma, as well as diabetes mellitus with disease-related ophthalmological findings and/or polyneuropathy. Systemic medication possibly influencing the PLR, such as anticholinergics, dopamine agonists, tricyclic antidepressants, antipsychotics, or antihistamines^26^ was another exclusion criterion.

Detailed history and ophthalmological examinations at the neuro-ophthalmological department of the University Eye Hospital Tuebingen, Tuebingen, Germany, included BCVA, refraction, optical coherence tomography (OCT; Spectralis-OCT; Heidelberg Engineering GmbH, Heidelberg, Germany; volume scan of the macular region and measurement of the peripapillary retinal nerve fiber layer [RNFL]), swinging-flashlight test, slit lamp examination, and fundus ophthalmoscopy. One eye without any exclusion criteria underwent 30 degrees static perimetry (Octopus 900, Haag-Streit International, Wedel, Germany).

### Chromatic Pupil Campimetry 

Hardware and Java-based software for the recording and real time calculation of the pupil diameter were custom-built at the Centre for Ophthalmology Tuebingen, Tuebingen, Germany.^1^

Recordings were performed in a dark, quiet room with an infrared camera (DMK23UV024, The Imaging Source GmbH; 50 mm TV lens 1:1.8; temporal resolution 10 ms) and a 55-inch OLED monitor (LG OLED 55C7V; LG Electronics, Seoul, South Korea; UHD 3840 × 2160 Pixel) at 40 cm distance to the participant's eye, the contralateral eye covered by an eye patch. A chin- and headrest assured stable fixation on a dim fixation mark (radius 1 degree, luminance 0.01 cd/m^2^) in the center of the monitor. The examiner's shielded screen was located beside the recording device for supervision and quality assurance.^2^

After individually adjusting the device, gaze tracking was calibrated ensuring retinotopic stimulation. Diverging fixation 10 ms before stimulus presentation led to automatic displacement of a stimulus. If the calculated stimulus position was located outside the monitor due to severe gaze deviation, the stimulus was repeated automatically later during the measurement.^1^

The subsequent stimulus protocols have been validated before.^2^^,^^13^^,^^17^^,^^20^^,^^25^ The photopic protocol was slightly adapted compared to former studies (increased number of stimulus repetitions per location for better accuracy, reduction of stimulus duration, and number of stimulus locations for shortening of recording time), both protocols contained an optimized latency calculation.

### Photopic CPC 

The photopic CPC (photCPC) protocol included a dim blue background (luminance 0.01 cd/m^2^; wavelength 460 ± 30 nm full width at half maximum [FWHM]; ≙2.1 × 10^−8^ W) for normalization of pupil size with certain rod saturation inducing the least possible pupillary constriction.^2^ Adaptation time to the background illumination during gaze calibration was approximately 2 minutes.

**Figure 1.**
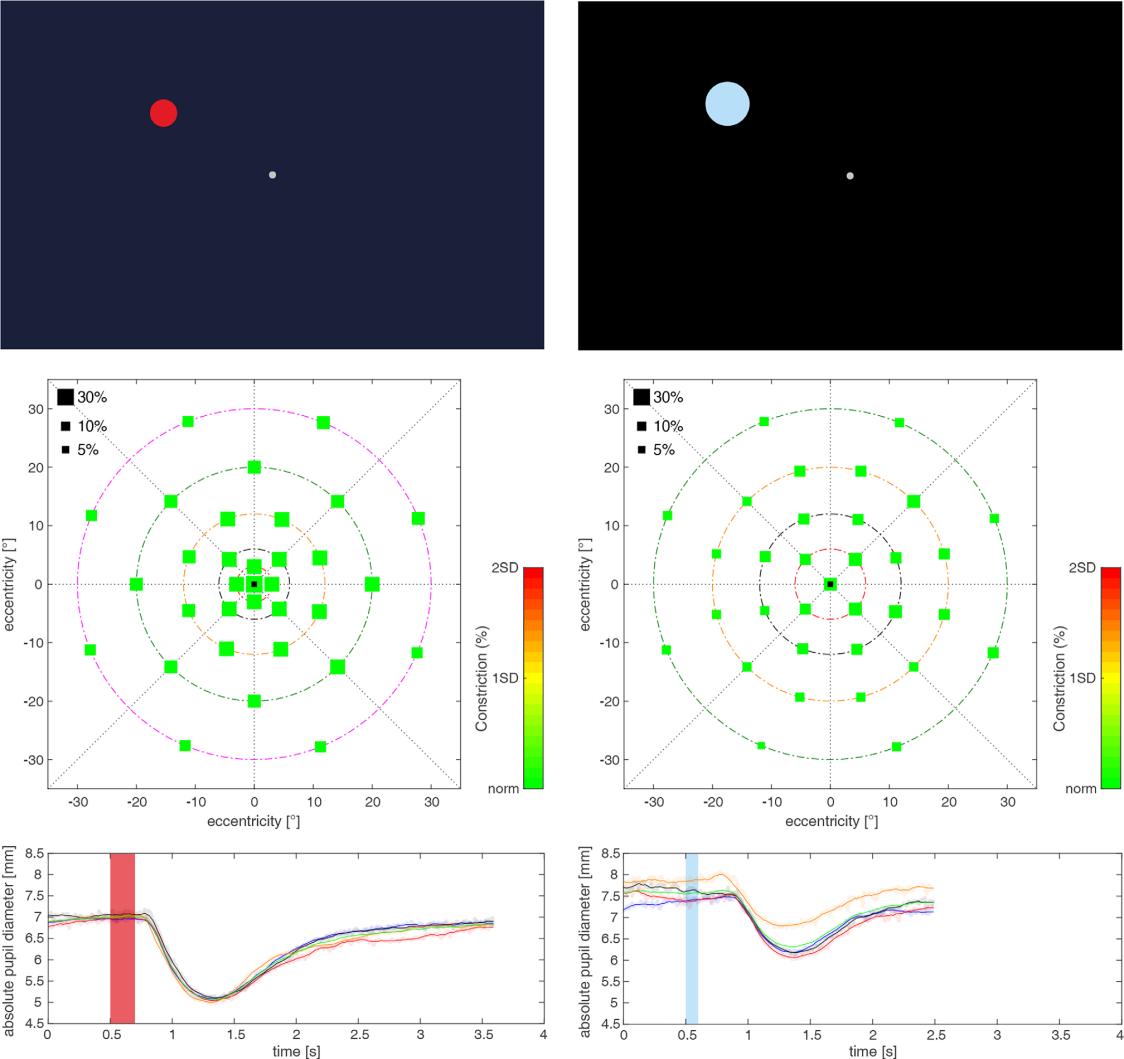
*Top*: Example of background illumination, fixation mark, and stimulus characteristics during photCPC (*left column*) and scotCPC (*right column*). *Middle*: Coordinate system in MATLAB containing the 33 stimulus positions, ID 031, right eye. Size of the squares indicates relative maximal constriction amplitude (%, relMCA), color represents deviation of relMCA from the mean of the study cohort (*green* = normal, *yellow* = −1 SD, and *red* = −2 SD). *Below*: Absolute pupil diameter (mm) as a function of time (seconds) is shown for the four repetitions (*orange*, *black*, *red*, and *blue*; *green curve* = mean) of the central stimulus (▪ in the coordinate system). Stimulus duration is indicated by the red (0.5–0.7 seconds) and blue (0.5–0.6 seconds) bar.

To stimulate L-cones as selectively as possible, red stimuli (radius 3 degrees, wavelength 620 ± 30 nm FWHM, luminance 60 cd/m^2^, irradiance 8.2 × 10^−4^ W/m^2^, peak intensity 0.0148 mW/m^2^; Fig. 1) were presented concentrically around the fixation point (0 degrees, 3 degrees, 6 degrees, 12 degrees, 20 degrees, and 30 degrees eccentricity) in a random order at 33 stimulus locations at four repetitions. The total time of one pupillogram was 3.7 seconds: 500 ms baseline + 200 ms stimulus duration + 3 seconds post stimulus interval. Not reaching 90% of the previous baseline pupil diameter after pupillary constriction or no pupillary signal, for example, due to severe blinking, for more than 300 ms within 1.5 seconds after stimulus onset led to automatic stimulus repetition. The total recording time of photCPC was approximately 8 minutes up to a maximum of 15 minutes in difficult recording conditions.

### Scotopic CPC 

After a 20-minute dark adaptation, the fixation mark and dim blue stimuli (radius 5 degrees, wavelength 460 ± 30 nm FWHM, luminance 0.01 cd/m^2^, irradiance 2.7 × 10^−5^ W/m^2^, peak intensity 7.52 × 10^−4^ mW/m^2^; see Fig. 1) were presented on a black background arranged slightly differently (0 degrees, 6 degrees, 12 degrees, 20 degrees, and 30 degrees eccentricity) compared to photCPC. The total time of one pupillogram was 2.6 seconds: 500 ms baseline + 100 ms stimulus duration + 2 seconds post stimulus interval and the total recording time was 6 to 10 minutes.

### Statistical Analysis

Pupil diameter during the PLR was charted and analyzed by a custom-made script in MATLAB R2020b (The MathWorks Inc., Natick, MA, USA; see Fig. 1). Every dataset was checked for an outright mean pupillogram per stimulus localization, individual curves were adjusted manually in case of blinking artifacts^2^ or removed when clearly defective.

Pupillary parameters of interest were baseline (mm, average pupil diameter during the 500 ms prior to stimulus presentation), latency (ms, time from stimulus onset to intersection of a linear fit through the descending part of the pupillogram with a linear fit through the baseline, preset range 150–700 ms), absolute maximal constriction amplitude (absMCA; mm, difference between baseline and lowest pupil diameter at maximal pupillary constriction, declared as noise and excluded automatically if <0.05 mm), and relative maximal constriction amplitude (relMCA; %, ratio of absMCA and baseline of one pupillogram according to the standards in pupillography^26^). Traces with amplitude or latency close to the cut-off (amplitude 0.05–0.1 mm, 200 ms > latency > 500 ms) were charted and checked for plausibility. The Hill of Vision as a three-dimensional illustration of the mean relMCA at every stimulus location within the tested 30 degrees VF serves as a visualization of certain pathologies affecting the pupillary constriction amplitude. The function volume ((degrees)^2^%, functional three-dimensional sum parameter of all relMCAs within 30 degrees eccentricity, enables concise comparison of cone and rod function in different studies) and angle (degrees, steepness angle of all relMCAs from 30 degrees eccentricity to the center, indicates the localization of a functional defect in the center (flatter angle),^20^ periphery (steeper angle), or throughout the retina) were calculated.

Statistical analysis (significance level α < 0.05) was performed with linear mixed-effects models in JMP 16.2 (SAS Institute, Cary, NC, USA), including all stimulus characteristics and clinical data with potentially statistically significant, clinically relevant effects on relMCA and latency (eccentricity, stimulus presentation in the temporal/nasal VF, upper/lower VF, daytime and season of the recording, age group, iris color, and gender). All parameters without statistically significant effect on relMCA or latency were removed. Remaining parameters were additionally interconnected as far as methodically permitted and analyzed with post hoc Tukey tests. Linear mixed-effects models including the applied post hoc Tukey tests were used to account for multiple testing. For determination of relMCA normative intervals per eccentricity, original relMCA data were transformed to logarithmic data because of slight deviation from normal distribution at 30 degrees eccentricity and subsequently transformed back to linear data.

A multivariate correlation analysis was conducted between the pupillary function volume within 0 degrees to 6 degrees eccentricity and OCT data (RNFL, macula volume, ganglion cell layer [GCL] volume, and outer retinal layer [ORL] volume; automatic thickness map calculation with a circle diameter of 3.45 mm).

To determine test-retest reliability, CPC recordings were performed twice within 3 months in a subgroup of 12 participants. The intraclass correlation coefficient (ICC) and Bland-Altman plots were calculated.

If not otherwise specified, results are outlined as mean ± standard deviation (SD) of the original data.

## Results

### Key Determinants of the PLR

**Figure 2.**
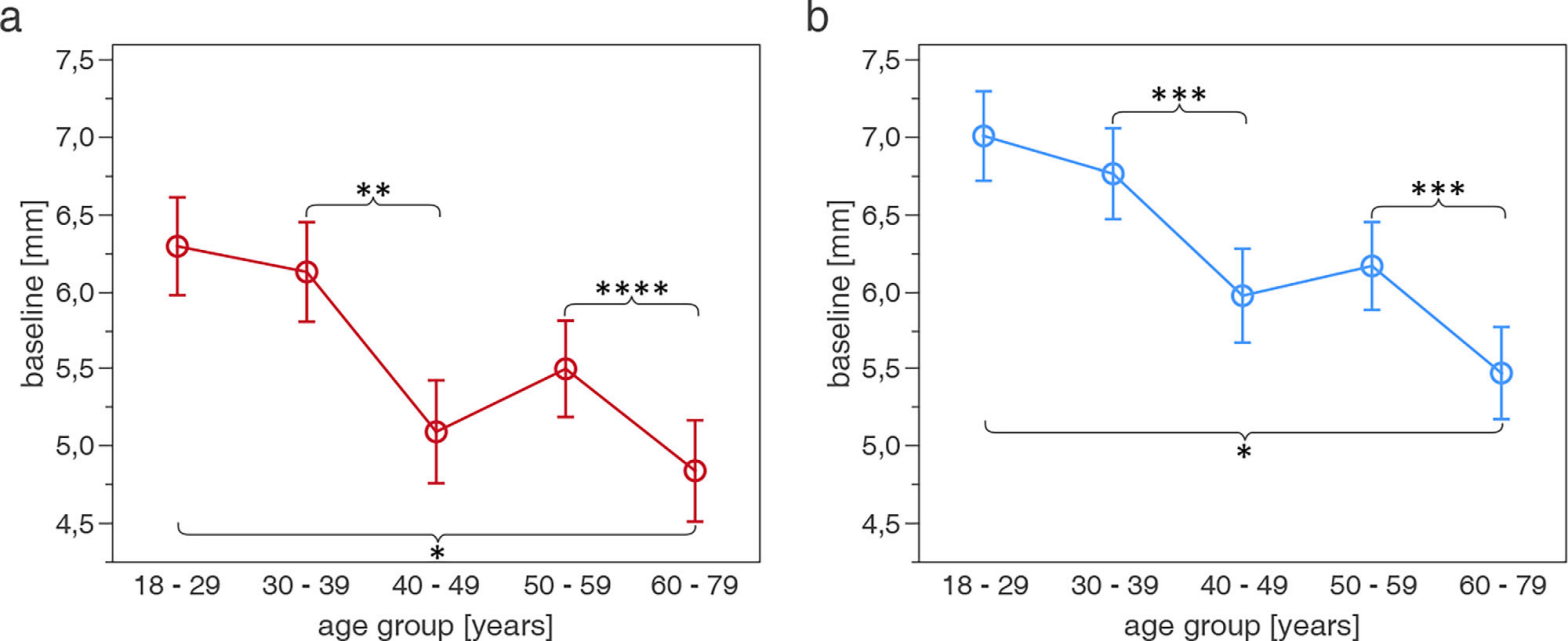
Significant decrease in baseline pupil diameter (mean ± confidence interval, mm) with increasing age (years) in photCPC (a) and scotCPC (b) (*n* = 150). *P < 0.0001, **P < 0.001, ***P < 0.01, ****P < 0.05. Figures are extracted from the linear mixed-effects model.

Mean baseline pupil diameter during photCPC was 5.59 ± 1.05 mm, thus smaller than in scotopic CPC (scotCPC) with 6.29 ± 0.98 mm. Baseline decreased statistically significantly with increasing age under both photopic and scotopic conditions (effect: *P* < 0.0001, respectively). A significant reduction was found between age groups 30 to 39 years and 40 to 49 years (difference in the linear mixed-effects model: 1.04 mm in photCPC [*P* = 0.0002]; 0.79 mm in scotCPC [*P* = 0.0029]), and between age groups 50 to 59 years and 60 to 79 years (difference photCPC: 0.66 mm [*P* = 0.0358]; scotCPC: 0.69 mm [*P* = 0.0098]). Compared with the youngest age group of 18 to 29 years, the baseline in the oldest age group of 60 to 79 years was 1.45 mm smaller in photCPC (*P* < 0.0001) and 1.53 mm smaller in scotCPC (*P* < 0.0001; Fig. 2).

Mean latency was shorter in photCPC (259 ± 35 ms) than in scotCPC (320 ± 32 ms). With increasing stimulus eccentricity, latency rose continuously from 244 ± 25 ms (central stimulus) to 278 ± 43 ms (30 degrees eccentricity) in photCPC and from 310 ± 26 ms (central stimulus) to 329 ± 36 ms (30 degrees) in scotCPC.

Mean absMCA was higher under photopic (0.67 ± 0.33 mm) than under scotopic conditions (0.58 ± 0.27 mm). Particularly, absMCA in the center was higher in photCPC (1.09 ± 0.31 mm) than in scotCPC (0.86 ± 0.27 mm). AbsMCA decreased with increasing eccentricity, which was more pronounced during photCPC, resulting in comparable absMCAs in the periphery in photCPC (0.41 ± 0.24 mm) and scotCPC (0.44 ± 0.25 mm). Age had no statistically significant effect on absMCA.

**Figure 3.**
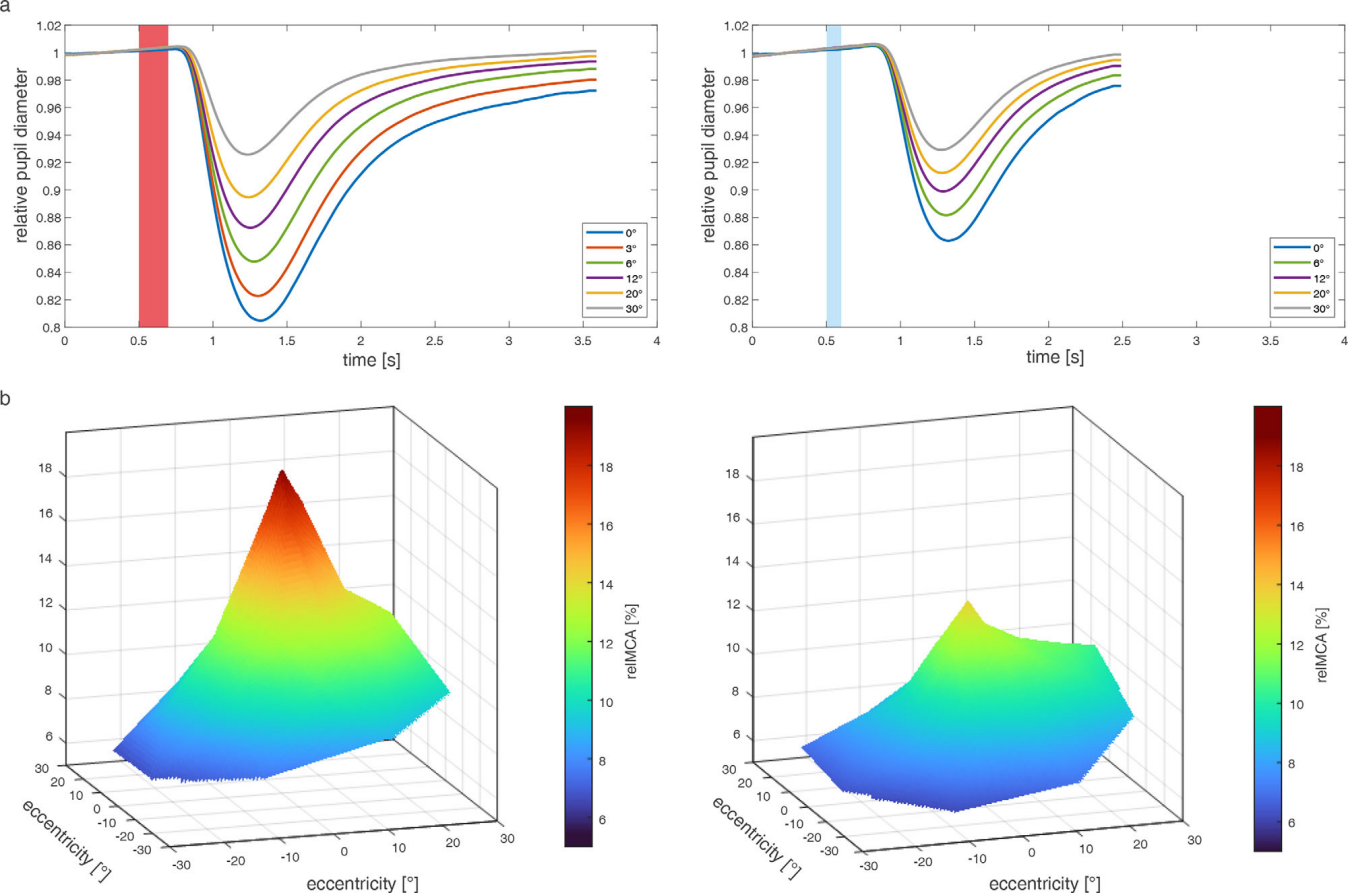
(a) Mean pupil diameter normalized to baseline (*n* = 150) decreasing with increasing stimulus eccentricity in photCPC (*left column*) and scotCPC (*right column*). Higher relative maximal constriction amplitude (relMCA) in the center at 0 degrees (*blue line*) during photCPC with comparable relMCAs at 30 degrees eccentricity in photCPC and scotCPC (*grey line*), leading to higher pupillary function volume and steepness angle of the “Hill of Vision” (b) in photCPC. Hill of Vision = topographic illustration of mean relMCAs (z-axis) per eccentricity (x- and y-axis). Data of all left eyes were mirror converted to right eyes for this graphic.

Overall relMCA was higher during photCPC (12.24 ± 5.97%) than during scotCPC (9.38 ± 4.62%). As for absMCA, relMCA showed a higher central peak in photCPC (19.79 ± 5.23%) with a stronger decline at increasing eccentricity (30 degrees: 7.61 ± 4.58%) than scotCPC (0 degrees: 13.87 ± 4.69%; and 30 degrees: 7.26 ± 4.18%; Fig. 3a), leading to a higher pupillary function volume and steepness angle of the “Hill of Vision” in photCPC (27,581 ± 10,399 (degrees)^2^%; 21.22 ± 5.48 degrees) than in scotCPC (22,961 ± 9,689 (degrees)^2^%; 12.15 ± 4.16; Fig. 3b).

### Influencing Factors on relMCA

Results in the next two sections are extracted from linear mixed-effects models.

**Figure 4.**
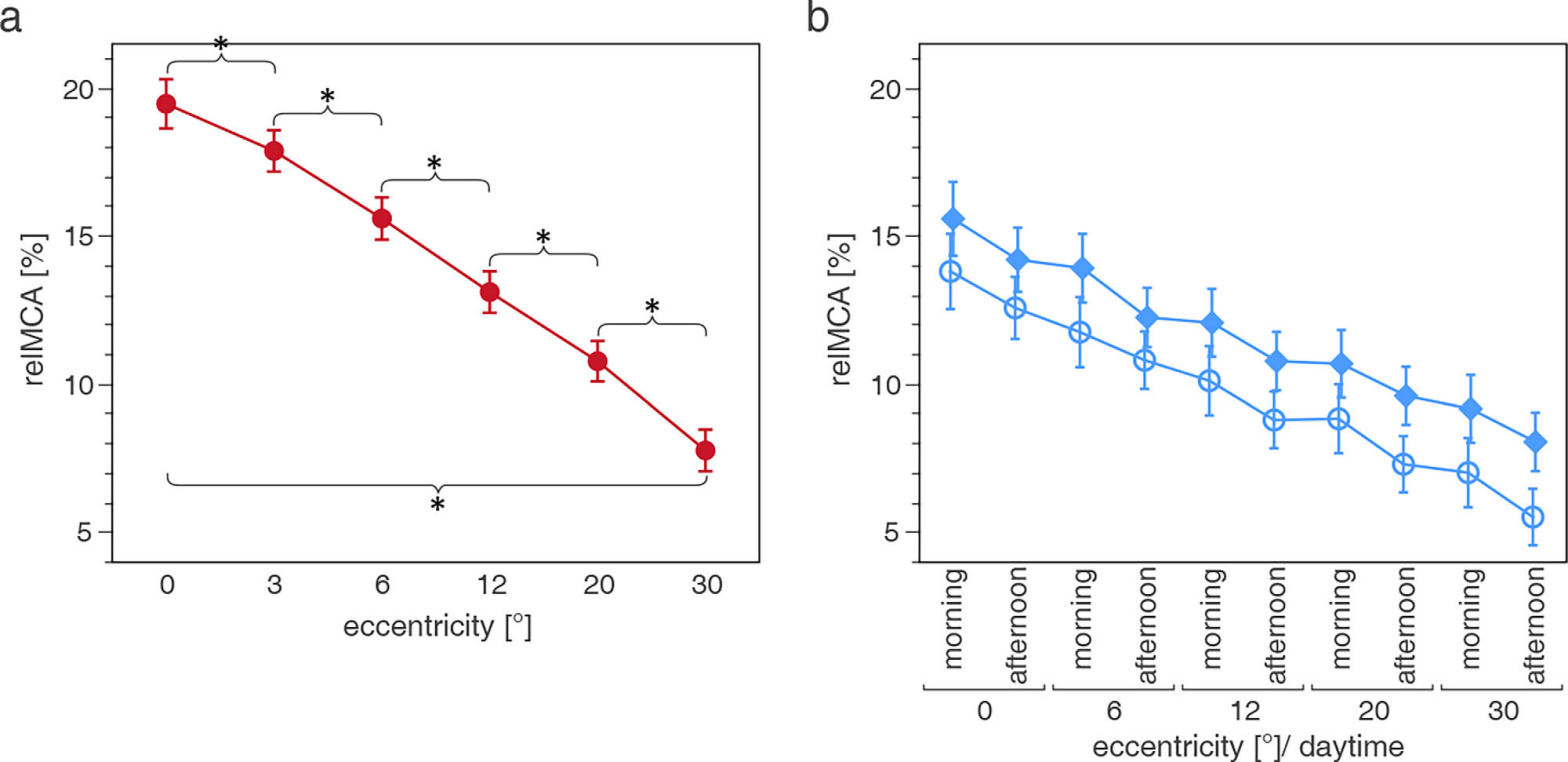
(a) PhotCPC: significant effect of eccentricity (degrees) on relative maximal constriction amplitude (relMCA; mean ± confidence interval, %). *P < 0.0001. (b) ScotCPC: significant effect of eccentricity (degrees), season (○ summer, ♦ winter), and daytime (morning and afternoon) on relMCA (mean ± confidence interval, %). For better clarity, no labeling of the significant decrease of relMCA with increasing eccentricity at measurements during the same season and daytime (*P* < 0.0001, respectively, *n* = 150). Figures are extracted from the linear mixed-effects model.

During photCPC, relMCA was influenced statistically significantly by eccentricity (effect of eccentricity on relMCA: *P* < 0.0001) and stimulus position in the temporal/nasal (*P* < 0.0001) and upper/lower VF (*P* < 0.0001). RelMCA declined significantly with increasing eccentricity (difference between adjacent eccentricities: 1.59–3.02%, *P* value post hoc Tukey test: *P* < 0.0001, respectively; Fig. 4), the difference between relMCA in the center and at 30 degrees was 11.71% (*P* < 0.0001). RelMCA in the temporal VF was slightly higher than in the nasal VF by 1.65% (*P* < 0.0001), and discreetly higher in the inferior than in the superior VF by 0.38% (*P* < 0.0001).

During scotCPC, eccentricity (*P* < 0.0001), season (*P* = 0.0013), and daytime (*P* = 0.0365), as well as the temporal/nasal (*P* < 0.0001) and upper/lower VF (*P* < 0.0001) significantly influenced relMCA. During measurements in the same season and daytime, relMCA decreased statistically significantly with increasing eccentricity (difference between adjacent eccentricities = 1.17–2.04%, *P* < 0.0001, respectively, e.g. 12 degrees, winter, morning > 20 degrees, winter, and morning; see Fig. 4). Mean relMCA was higher in winter than in summer and in the morning higher than in the afternoon, although not statistically significant when compared at individual eccentricities. As during photCPC, relMCA under scotopic conditions was slightly higher in the temporal than in the nasal VF (difference = 1.46%, *P* < 0.0001), but discreetly higher in the superior than in the inferior VF (difference = 0.45%, *P* < 0.0001). The age group did not have a statistically significant effect on relMCA in photCPC or scotCPC.

### Influencing Factors on Latency

**Figure 5.**
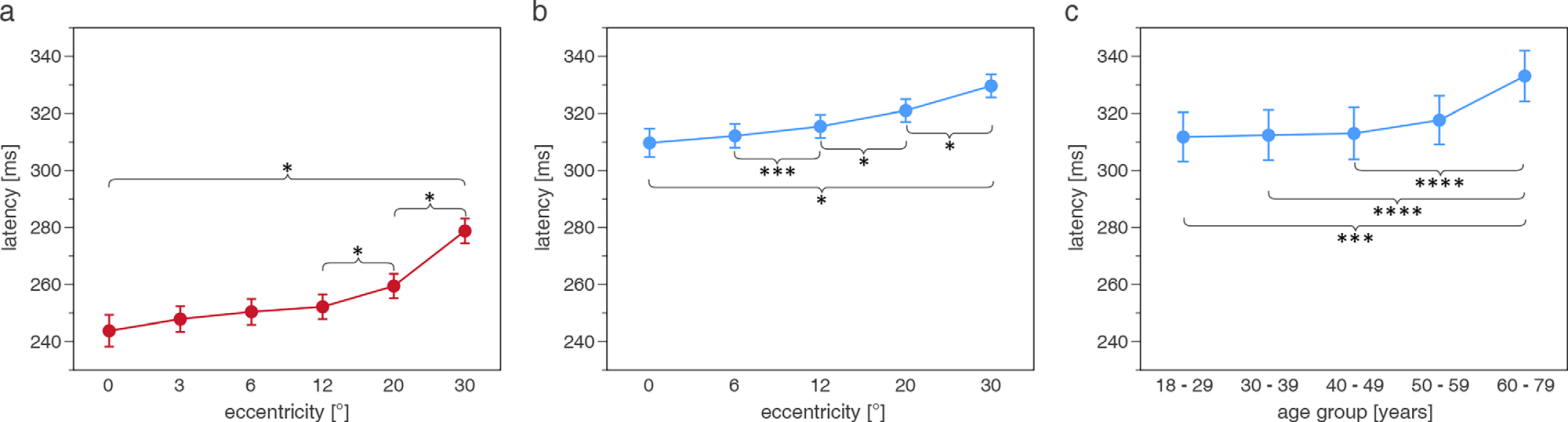
(a) PhotCPC: significant effect of eccentricity (degrees) on latency (mean ± confidence interval, ms). ScotCPC: significant effect of eccentricity (degrees) (b) and age group (years) (c) on latency (mean ± confidence interval, ms) (*n* = 150). *P < 0.0001, ***P < 0.01, ****P < 0.05. Figures are extracted from the linear mixed-effects model.

During photCPC, a significant effect on latency was found for eccentricity (*P* < 0.0001; Fig. 5) and the temporal/nasal VF (*P* < 0.0001). Latency rose with increasing eccentricity, statistically significantly from 12 degrees to 20 degrees (difference = 7 ms, *P* < 0.0001) and 20 degrees to 30 degrees (difference = 19 ms, *P* < 0.0001) with a difference between the center and periphery of 35 ms (*P* < 0.0001). Latency was slightly longer in the nasal than in the temporal VF (difference = 13 ms, *P* < 0.0001).

Under scotopic conditions, latency was influenced significantly by eccentricity (*P* < 0.0001) and age group (*P* = 0.0043; see Fig. 5). As in photCPC, latency increased with increasing eccentricity, statistically significantly from 6 degrees to 12 degrees (difference = 3 ms, *P* = 0.0066), 12 degrees to 20 degrees (difference = 6 ms, *P* < 0.0001), and 20 degrees to 30 degrees (difference = 9 ms, *P* < 0.0001). Latency at 30 degrees was 20 ms longer than in the center (*P* < 0.0001). Age groups 18 to 29 years, 30 to 39 years, and 40 to 49 years showed similar latencies. Latency rose statistically significantly between age groups 18 to 29 years (30–39 and 40–49 years) and 60 to 79 years by 21 ms (21 and 20 ms) (*P* = 0.0078, *P* = 0.0118, and *P* = 0.0186, respectively) in scotCPC.

### Parameters Without Effect on PLR

The participant's iris color and gender did not influence relMCA or latency in L-cone- or rod-favoring stimulation. No relevant correlation was found between the pupillary function volume and OCT data RNFL, macula volume, GCL, and ORL volume. Only under photopic conditions, there was a statistically significant but weak correlation of 0.25 between RNFL and 0 degrees to 6 degrees volume (*P* = 0.0024).

### Test-Retest Reliability

RelMCA calculation showed excellent test-retest reliability in cone- (ICC = 0.92, Bland-Altman = 0.92, mean difference of relMCA between the two measurements = 0.72%, 95% confidence interval on the limits of agreement [LOA] = −4.87 to 6.30%) and rod-protocols (ICC = 0.86, Bland-Altman = 0.87, mean difference = 0.94%, and LOA = −4.43 to 6.32%). Moderate to poor test-retest reliability was found for latency (photCPC ICC = 0.53, Bland-Altman = 0.54, mean difference = 5 ms, LOA = −48 to 59 ms; scotCPC ICC = 0.25, Bland-Altman = 0.25, mean difference = 2 ms, and LOA = −58 to 63 ms).

### Normative Database for CPC

Based on the beforementioned results, a normative database for photCPC and scotCPC was established including relMCA (Table 1) and latency (Table 2) per eccentricity, pupillary function volume, and steepness angle (see Table 1). Normative values for latency under scotopic conditions are provided separately for age <60 versus ≥60 years.

**Table 1.** Normative Data for Relative Maximal Constriction Amplitude (relMCA, %) per Eccentricity, Pupillary Function Volume ((degrees)^2^%), and Steepness Angle (degrees) in photCPC and scotCPC With Mean and 95% Normative Interval

	relMCA (%) PhotCPC		relMCA (%) ScotCPC	
Eccentricity	Mean	95% Normative Interval	Mean	95% Normative Interval
0 degrees	19.02	**10.65–33.96**	12.96	**5.98–28.09**
3 degrees	17.15	**9.12–32.25**	n.a.	n.a.
6 degrees	14.56	**7.10–29.86**	11.05	**4.61–26.46**
12 degrees	11.95	**5.12–27.91**	9.19	**3.34–25.28**
20 degrees	9.52	**3.19–28.39**	7.78	**2.49–24.31**
30 degrees	6.08	**1.30–28.46**	5.83	**1.27–26.81**
**Pupillary function volume (degrees)^2^%**				
	27,581	7,199–47,963	22,961	3,971–41,951
**Steepness angle (degrees)**				
	21.22	10.26–32.18	12.15	3.83–20.47

Bold values indicate 95% normative intervals for relMCA.

**Table 2.** Normative Data for Latency (ms) in photCPC and scotCPC per Eccentricity and Age <60/≥60 Years in scotCPC With Mean and 95% Normative Interval

	Latency (ms) PhotCPC		Latency (ms) ScotCPC			
Eccentricity	Mean	95% Normative Interval	Mean		95% Normative Interval	
			<60 y	≥60 y	**<** **60 y**	**≥** **60 y**
0 degrees	244	**196–291**	306	324	**258–354**	**274–374**
3 degrees	248	**195–300**	n.a.	n.a.	n.a.	n.a.
6 degrees	250	**196–305**	308	328	**258–358**	**273–383**
12 degrees	252	**195–309**	312	331	**258–365**	**272–390**
20 degrees	259	**193–325**	317	337	**259–375**	**272–402**
30 degrees	278	**189–366**	326	342	**259–392**	**262–421**

Bold values indicate 95% normative intervals for latency.

## Discussion

This is the first study in which CPC was performed in a large cohort of 150 healthy participants to investigate influencing factors on the PLR and to provide further test quality criteria by implementing normative data. Meanwhile, CPC is frequently used to objectively measure cone and rod function as outcome parameters in gene therapy studies,^13^ in the natural course of retinal degenerations^17^^,^^20^ and the investigation of the pupillary pathway.^25^ Therefore, a normative database including relevant impact factors on the PLR is not only crucial for the evaluation and comparability of CPC results in the clinical setting but likewise relevant for the entire field of pupil research.

The reduction in baseline pupil diameter from the youngest to the oldest age group of 1.45 mm in photCPC and 1.53 mm in scotCPC is in accordance with previous findings of an average of approximately 0.04 mm decrease per year under weak illumination.^27^^,^^28^ This validates our method as precise enough to reproduce such expected effects and serves as a proof-of-concept for a reliable evaluation of pupillary responses and possible influencing factors. It additionally emphasizes the importance of the normalization of the pupillary constriction amplitude to the baseline pupil diameter in PLR evaluation.

Few studies have shown a statistically significant effect of age on the PLR at high irradiances despite normalization to the baseline pupil diameter.^29^^,^^30^ However, an age-independent preservation of the cone- and rod-mediated transient PLR and maximal relative constriction amplitude as well as the post-illumination pupil response (PIPR) mediated by melanopsin sensitive intrinsically photosensitive retinal ganglion cells (ipRGCs)^31^ could also be demonstrated.^32^ Different stimulation characteristics^33^ and age categorization are plausible reasons for the inconsistency of these results. In our study, age group did not have a statistically significant effect on absMCA or relMCA and not on latency during photCPC. Yet, we observed an interesting increase of around 20 ms in latency under scotopic conditions in the oldest age group (60-79 years). It has been shown that there is a reduction in photoreceptor count and sensitivity with age, especially in rods.^34^ However, this aging process was not reflected in the constriction amplitude in CPC, wherefore no age correction is necessary in the normative database for relMCA. In a previous study on rods,^3^ we likewise showed that the PLR through suprathreshold stimulation in CPC is not correlated with retinal sensitivity measured by dark-adapted chromatic perimetry, confirming the complementary benefit of both methods in the functional diagnostics of rods. Due to the suprathreshold stimulation, CPC in healthy participants is considered as a measure of the number of functioning photoreceptors and the functioning of the subsequent neuronal network. Certainly, more intense stimuli produce likewise more pronounced pupillary responses and reflect the sensitivity of the retinal system, but CPC is not designed for photoreceptor sensitivity threshold determination. CPC is suitable to detect PLR changes in variable diseases ranging from photoreceptor dysfunction (RP,^35^ AMD,^20^ and hereditary macular dystrophies^17^) to hemianopic VF defects in occipital ischemia.^23^ Even the varying loss of photoreceptor function in different genotypes of RP^35^ and effects of retinal gene therapy can be detected by CPC.^13^ Yet it remains unclear to what extent CPC can detect the loss of a certain number of cells in the healthy aging retina despite possible compensatory mechanisms within the retina. Very subtle loss of photoreceptor sensitivity might not be detected due to the suprathreshold stimulation in pupil research and interindividual variability of the PLR. Furthermore, there is the impact of summation and convergence of multiple photoreceptors to ipRGCs. Hence, the combined analysis of relMCA and latency in patient data seems favorable.

The two stimulation protocols applied in this study were found to be suitable for the specific and retinotopic evaluation of predominantly either cone or rod function, respectively. The distribution pattern of functioning retinal photoreceptors can be examined by relMCA as depicted in the “Hill of Vision” with a high central peak in photCPC correlating with high cone density in the fovea in the healthy human retina.^36^^,^^37^ The small central peak of relMCA in scotCPC can be explained by a spatial filtering model including the stimulus size, which converts the hill shaped distribution pattern of rod responses into the ring-structure of retinal rod density.^3^ Peak rod density is located at 15 degrees to 20 degrees whereas rod bipolar density is highest at 7 degrees to 15 degrees and AII amacrine cell density highest at 2 degrees to 7 degrees.^38^ The changing summation effects from the central to the peripheral retina in the rod pathway, rod convergence, and lower spatial resolution due to large scotopic stimuli result in higher central rod responses in CPC.

Of the numerous epidemiological and ophthalmological data, stimulus eccentricity was found to have the greatest impact on relMCA and latency of the PLR during L-cone- and rod-favoring stimulation, which is consistent with previous studies.^2^^,^^39^^–^^41^ Retinal photoreceptor distribution, as mentioned before, as well as decreasing ganglion cell density in the retinal periphery^42^ and subsequently lower activation of the pupillary pathway can be discussed as possible reasons.^39^

Considering the present large study collective, it can be postulated that the PLR is independent of iris color and gender. Confirmatively, iris color did not significantly affect the relative constriction amplitude^43^ and both constriction amplitude and latency were not influenced significantly by gender^44^ in previous studies. OCT data are not relevantly correlated with the pupillary function volume in CPC in a normative cohort. This highlights that the morphological volume of individual retinal layers in the healthy human retina does not directly influence the extent of pupillary constriction amplitudes which fits to the observed morphological-functional gap seen in retinal degeneration.^35^^,^^45^

The statistically significant effects of VF, season, and daytime on relMCA and latency for certain stimulus conditions are considered as not clinically relevant but will be shortly discussed for scientific purpose and completeness. Stimulation in the temporal visual hemifield resulted in subtle relMCA elevation in photCPC and scotCPC and discreetly shorter latencies in photCPC compared with the nasal VF. A limitation for temporal-nasal interpretation is the centered position of the participant´s head in front of the monitor and the examined eye consequently positioned slightly temporally which might discreetly accentuate the temporal hemifield. Under photopic conditions, relMCA was slightly more pronounced inferiorly than superiorly and vice versa under scotopic conditions. In a previous study with different stimulation characteristics, the temporal superior VF was found to be the most sensitive.^46^ The lower hemifield has been shown to be particularly sensitive for stimulus discrimination, especially color discrimination,^47^ that is, for activities during photopic conditions with cone involvement. However, the measured differences are of such small extent and close to the limit of error range that they can be considered negligible in PLR evaluation.

Only relMCA under scotopic conditions was influenced by season and daytime with higher mean relMCA in winter and morning measurements. These differences were not statistically significant when compared at individual eccentricities and thus not further investigated. Münch et al.^48^ showed that pupillary responses driven by ipRGCs are higher in winter whereas the cone- and rod-mediated PLR is independent of the season. The PIPR by ipRGCs was found to alter significantly throughout the day in correlation with the circadian changes in melatonin levels, peaking around 9:30 PM, whereas cone responses did not change significantly within the day.^49^

Excellent test-retest reliability could be shown for relMCA and confirms its suitability as a robust biomarker in clinical application. Test-retest reliability of latency was moderate for photopic stimulation and relatively poor for scotopic stimulation. Although the mean differences in test-retest latency calculation were marginal and methodologically in the range of latency calculation precision limit, there was a substantial scattering in the differences of individual measurements, especially for small PLR. We consider two factors: more intra-individual variability of latency^50^ and small inaccuracies in latency calculation as it is much more demanding as relMCA calculation, particularly for small amplitudes.

To conclude, the current study demonstrates the robustness of CPC, validating relMCA and latency as suitable readout parameters. The presented normative database enables an easily applicable classification of patients’ results in CPC considering stimulus eccentricity as the relevant impact factor on the PLR. Hereby, an essential prerequisite for the use of relMCA and latency of the PLR as biomarkers for local retinal function has been established.

### Limitations

Even though a high number of participants has been included in this study, the analysis of relMCA showed a high variability particularly at peripheral stimulus positions. However, conclusive normative intervals in the periphery are crucial especially for the evaluation of photoreceptor function in patients with RP. Since the very beginning of clinical pupil research, a high interindividual variability of PLR parameters has been described. As other factors have been ruled out, we propose this natural variability to be the main reason for the broad standard deviations observed in our trial. It limits the interpretation of an isolated, single CPC measurement in individual patients. Yet, in combination with other biomarkers, CPC's diagnostic value is relevant. In clinical practice, the interindividual variability may be less important than the intra-individual one: the primary application of CPC will be the follow-up of disease progression or as a functional biomarker in interventional clinical trials.
